# Toxic Metals and Metalloids in Food: Current Status, Health Risks, and Mitigation Strategies

**DOI:** 10.1007/s40572-024-00462-7

**Published:** 2024-10-01

**Authors:** Di Zhao, Peng Wang, Fang-Jie Zhao

**Affiliations:** College of Resources and Environmental Sciences, Nanjing Agricultural University, Nanjing, 210095 China

**Keywords:** Metals/metalloids, Dietary intake, Contamination status, Risk assessment, Intervention strategies

## Abstract

**Purpose of Review:**

Exposure to toxic metals/metalloids, such as arsenic (As), cadmium (Cd), and lead (Pb), through food consumption is a global public health concern. This review examines the contamination status of these metals/metalloids in food, assesses dietary intake across different populations, and proposes strategies to reduce metal/metalloid exposures throughout the food chain.

**Recent Findings:**

For the general population, dietary intake of metals/metalloids is generally lower than health-based guidance values. However, for vulnerable populations, such as infants, children, and pregnant women, their dietary intake levels are close to or even higher than the guidance values. Among different food categories, seafood shows higher total As, but largely present as organic species. Rice accumulates higher As concentration than other cereals, with inorganic As (iAs) and dimethylarsinic acid (DMA) being the main As species. Methylated thioarsenate species, such as dimethylmonothioarsenate, have also been detected in rice. The distribution of iAs and DMA in rice shows geographical variation. Additionally, seafood and cocoa products generally contain more Cd than other food, but seafood consumption does not significantly increase in adverse health effects due to its high zinc and iron content. Compared to As and Cd, Pb concentrations in food are generally lower. To minimize the health risks of metal/metalloid exposure, several strategies are proposed.

**Summary:**

Food contamination with toxic metals/metalloids poses significant concerns for human health, particularly for vulnerable populations. This review provides scientific evidence and suggestions for policy makers to reduce human exposure of metals/metalloids via dietary intake.

## Introduction

Metals are naturally occurring elements in the Earth’s crust and can be found in soil, water, and air. They include essential metals, which are crucial for various biological processes, and non-essential metals, such as cadmium (Cd) and lead (Pb), which can be toxic even at relatively low concentrations. Additionally, toxic metalloids such as arsenic (As) are increasing in the environment due to inputs from natural sources and human activities. These metals and metalloids have attracted extensive attention since they are top hazardous substances listed on the US Agency for Toxic Substances and Disease Registry (ATSDR)’s 2022 Priority List [1]. Besides being classified as known or probable human carcinogens by the International Agency for Research on Cancer (IARC), studies have also shown that these metals/metalloids are associated with numerous adverse health effects [2]. Specifically, As exposure has been linked to skin hyperkeratosis, increased risk of cardiovascular disease, diabetes, and respiratory failure [3]. Cadmium exposure can be particularly harmful to renal injury, while Pb exposure can impact the nervous system, especially for children, thereby raising concerns about their potential adverse effects on human health [4, 5].

Human exposure to toxic metals/metalloids can occur through various routes, including ingestion of contaminated food, drinking water, inhalation/ingestion of dust, and dermal contact. In areas where As occurs naturally at high levels in groundwater, drinking water is the primary source of As exposure [6]. However, for populations with lower levels of As in drinking water, food becomes the main route of As exposure [7, 8]. For the non-smoking population, dietary intake is also the primary source of Cd exposure, accounting for ~ 90% of the total exposure [9]. For Pb, food is the main source of exposure, although inhalation/ingestion of dust can also contribute, particularly for children [10]. Therefore, food contamination with toxic metals/metalloids pose significant concerns for both food safety and human health.

Although toxic metals/metalloids can occur naturally, human activities such as mining/smelting, irrigation with contaminated groundwater, atmospheric deposition, and the use of metal-containing fertilizers, manures, sewage sludge, and pesticides have contributed to environmental metal/metalloid pollution [11–14]. As a result, food produced in these polluted areas often contains elevated levels of metals/metalloids, exceeding the food safety limits and posing significant risks to human health [15–18].

In this review, we focus on recent progress in understanding the contamination status of three toxic metals/metalloids, including As, Cd, and Pb in food. Additionally, it addresses the potential health risks associated with metal/metalloid exposures through food consumption, and highlights the primary sources of food categories that contribute the most to overall dietary exposure. Finally, strategies to reduce metal/metalloid exposures throughout the food chain are also briefly discussed.

## Concentrations of Toxic Metals/Metalloids in Food

### Arsenic in Food

Arsenic poisoning is widespread in many countries due to contamination of As in drinking water, especially in Asia and South America [6, 19]. To protect humans from As exposure through drinking water, the World Health Organization (WHO) has established a guideline value of 10 µg/L for As in drinking water. Globally, an estimated 94 to 220 million individuals may be potentially exposed to unaccepted levels of As (> 10 µg/L) in the drinking water [6]. However, both animal and epidemiological studies have shown that chronic exposure to drinking water containing 10 µg/L of As can lead to various adverse health effects. For example, a recent mouse bioassay revealed that male mice exposed to drinking water with 10 µg/L of As for 6 months experienced reduced weight gain due to alterations in gut microflora and intestinal inflammation [20]. Additionally, a systematic review of 30 years of epidemiological evidence indicated that exposure to 10 µg/L of As in drinking water may increase the risk of bladder cancer by 40% compared to unexposed populations [21].

**Fig. 1:**
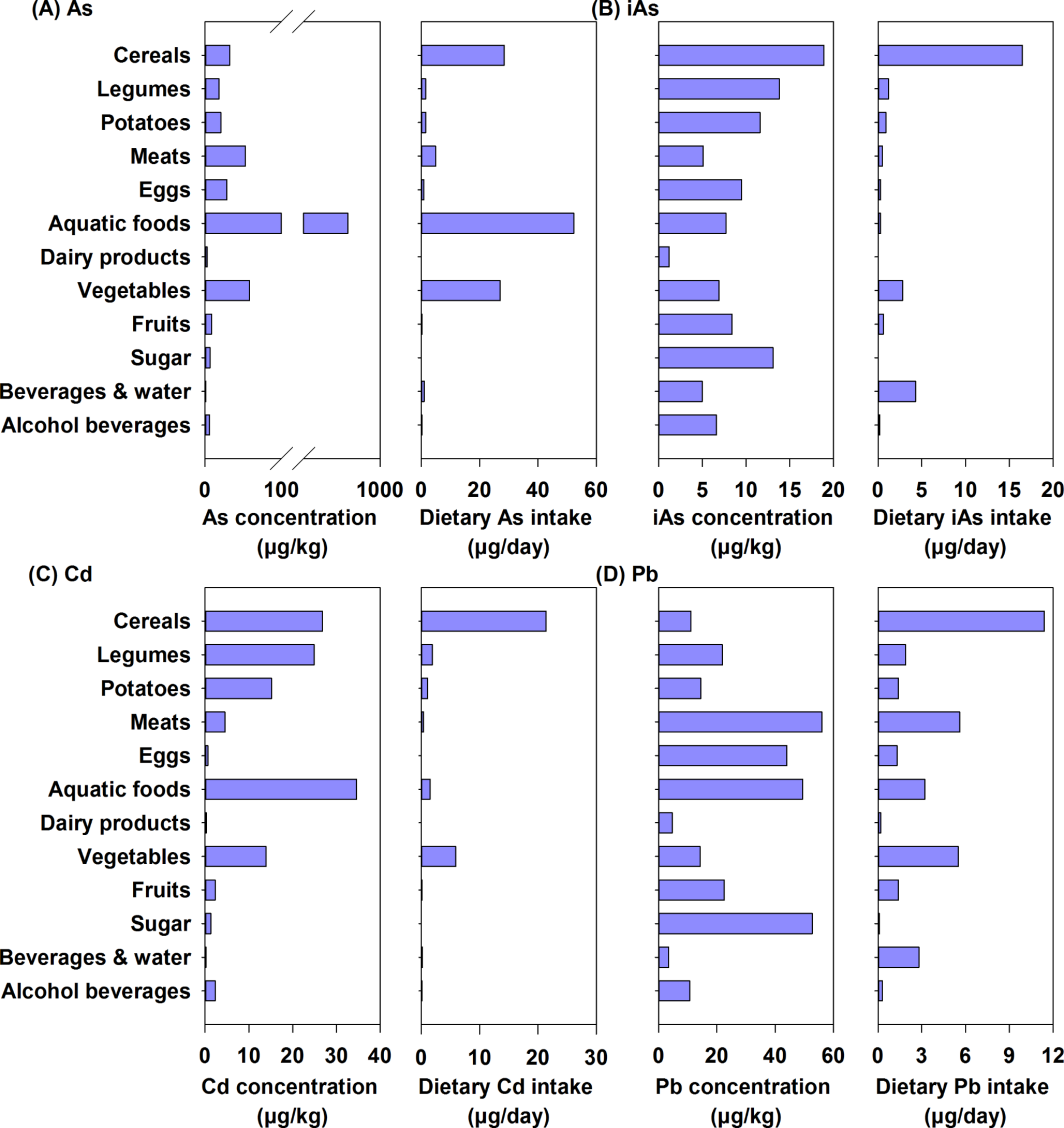
The mean concentrations of metals/metalloids in food and their dietary intake for Chinese adults (data obtained from [24])

Where As concentration in the drinking water is not elevated, As exposure from food becomes the dominant source [22, 23]. The ranges of total As concentration in different food categories collected from Australia, Brazil, France, Japan, New Zealand, and Singapore are shown in Table 1. Overall, wide ranges of As concentrations have been reported in different food categories from various countries, ranging from 0.001 mg/kg to 236 mg/kg (fresh weight) (Fig. 1A). Seafood, including seaweed, fish, and shellfish/molluscs, contains higher As concentrations compared to other food categories, with As concentrations in dried seaweeds, fish, and shellfish/molluscs being 0.114–236 mg/kg, 0.10–62 mg/kg, and 0.09–66 mg/kg, respectively (Table 1). Recent Total Diet Study conducted in Chile, China, and Japan reported similar As concentrations in seafood compared to those observed in this study [16, 24, 25]. Even though seafood exhibits the highest total As among different food groups, As is present primarily as organoarsenicals, which are considered to be less toxic compared to inorganic As (iAs) [26]. Specifically, arsenobetaine (AB) is the major form of As in most finfish and shellfish, although more complex organic As compounds such as arsenosugars and arsenolipids are also found in significant quantities in certain seafood products [26]. Increasing evidence indicates that some organic As species and their intermediate metabolites exhibit cytotoxicity in cell cultures [27, 28]. Therefore, future studies focusing on the potential health hazards of organic As species are needed. Moreover, some seafood contains substantial amounts of iAs. For example, the seaweed hijiki (*Hizikia fusiforme*), commonly consumed in Japan and Korea, has average concentrations of total As and iAs at 110 mg/kg and 77 mg/kg, respectively [29]. Blue mussels such as *Mytilus edulis* also contain relatively high concentrations of iAs, ranging from 0.001 mg/kg to 4.5 mg/kg [30].

**Table 1:** Occurrence data of total As, Cd, and Pb in food summarized by food category

Food category	As			Cd			Pb	
	No.	Range of total As concentrations (mg/kg)		No.	Range of national mean Cd concentrations (mg/kg)		No.	Range of national mean Pb concentrations (mg/kg)
Alcoholic beverages	462	0.001–0.05		3443	ND–0.004		2304	ND–0.38
Animal & vegetable fats	39	0.003–0.18		1610	ND–0.006		102	ND–0.002
Baked foods	71	0.002–0.25		55	ND–0.02		203	0.001–0.23
Beverages	120	0.001–0.26		3932	ND–0.003		4426	ND–0.35
Cereals	410	0.007–0.43		12,637	ND–0.02		5027	ND–0.029
Coffee & tea & cocoa	–	–		3505	0.0001–1.8		764	ND–1.03
Dairy products	376	0.001–0.35		9208	ND–0.004		3833	0.001–0.013
Dried fruit	–	–		79	0.003–0.009		282	0.006–0.34
Dried seaweeds	953	0.114–236		–	–		–	–
Eggs	171	0.003–0.04		736	0.0001–0.007		785	ND–0.039
Fish	1409	0.10–62		10,531	ND–0.008		656	ND–0.22
Fruits	966	0.005–2.2		6314	0.001–0.007		7480	ND–0.13
Meat muscle	4977	0.004–0.78		1715	0.001–0.003		1817	0.0001–0.013
Mushrooms and fungi	302	0.011–5.79		–	–		–	–
Nuts & oilseeds	70	0.005–0.88		350	0.02–0.1		184	ND–0.024
Oats	–	–		211	0.003–0.02		63	ND–0.003
Offal	2074	0.009–0.45		1406	0.03–0.5		73	0.006–0.042
Poultry offal	–	–		1224	0.006–0.5		1589	0.003–0.021
Pulses & legumes	–	–		169	0.003–0.03		326	ND–0.06
Rice	1693	0.002–1.83		2295	0.004–0.02		85	ND–0.004
Roots & tubers	–	–		2319	0.006–0.04		1255	0.001–0.065
Shellfish/molluscs	171	0.09–66		7403	0.01–4.8		765	0.01–0.19
Spices	–	–		2237	0.006–0.2		86	ND–0.11
Sugar & honey	138	0.003–0.26		3908	ND–0.03		1962	ND–0.082
Vegetables	2503	0.001–1.27		18,183	0.006–0.1		13,402	ND–0.4
Wheat	–	–		1503	0.009–0.04		506	ND–0.009

Data obtained from [143, 144]. ND refers to not detected.

In addition to seafood, mushrooms and fungi are also found to contain high levels of total As (0.01–5.79 mg/kg) (Table 1). Li et al. [31] found similar total As concentrations in 141 mushrooms from markets in 9 capital cities across China, ranging from 0.01 to 8.31 mg/kg. Of the 141 samples, 16% exceeded the As limit (0.5 mg/kg) for mushrooms in China. In comparison, As concentrations in mushrooms cultivated in Bangladesh (0.18–0.91 mg/kg) were lower [32]. The large variation in mushroom As concentrations may be attributed to the mushroom species. For example, *Laccaria vinaceoavellanea* showed a higher ability to accumulate As compared to other mushroom species, with As concentrations in the caps reaching 163 mg/kg despite the soil for cultivation containing only 5.6 mg/kg As [33]. Similarly, among 9 mushroom species collected from markets, *Agaricus bisporus*,*Pleurotus ostreatus*, and *Lentinula edodes* were found to have significantly higher total As concentrations than other species [31].

Additionally, elevated As concentrations have been reported in cereal and cereal products (0.007–0.43 mg/kg), rice and rice products (0.002–1.83 mg/kg) (Table 1). Rice accumulates approximately 10 times higher As than other cereals due to the enhanced As bioavailability under flooded conditions and the inadvertent yet efficient uptake of arsenite through the silicon pathway in rice roots [34, 35]. For 901 polished rice samples collected from 10 countries, median total As concentration varied 7 times, with the US (0.25 mg/kg) and France (0.28 mg/kg) having the highest median As concentration, while Egypt (0.04 mg/kg) having the lowest median As concentration [8]. Unlike seafood, the main As species in rice are iAs and dimethylarsinic acid (DMA), with monomehtylarsonic acid (MMA) being detected at low proportions occasionally. Since DMA and MMA are assumed to be less toxic to humans than iAs, more attention has been paid to iAs in rice. For a large survey conducted in 29 countries, iAs concentrations varied from < 0.002 mg/kg to 0.39 mg/kg in a total of 1180 polished rice samples, with 0.4% of rice samples exceeding the maximum level (0.2 mg/kg) set for iAs in rice [36]. Whereas for infants, the concentrations of iAs found in the global survey are problematic, since about 17.6% of the rice samples exceed the standard set for infants (0.1 mg/kg), highlighting the potential health risks of As exposure for vulnerable populations.

Among different regions around the world, the concentration of iAs in rice varies considerably, with rice from South America containing relatively high levels of iAs [36]. Similarly, the proportion of DMA concentrations in rice varies geographically. Studies have shown higher concentrations of DMA, as well as higher proportions of DMA in the total As in rice from North America and Europe than from Asia [8, 37, 38]. Rice produced in different regions of China also shows geographical variation in As species, with those produced in northeast China generally containing more DMA than in other regions [39]. Recently, methylated thioarsenate species, such as dimethylmonothioarsenate (DMMTA), have been detected in rice and rice products [40, 41]. DMMTA is known to be much more cytotoxic to human cell lines than iAs [42]. Importantly, the routine acid-based extraction and analysis of As species in rice converts the highly toxic DMMTA to relatively nontoxic DMA [40, 41]. In a global market-basket survey, the concentration of DMMTA-As in rice ranged from < 0.0002 to 0.035 mg/kg [41]. Further studies are needed to assess the health risk of DMMTA in rice.

## Cadmium in Food

Food intake is the predominate pathway for human Cd exposure. The ranges of Cd concentration in food covering 19 European countries as well as 11 other countries including Australia, Brazil, Canada, Chile, China, France, Ghana, Japan, Singapore, the US, and Vietnam are shown in Table 1. National average Cd concentrations in different food categories range from below detection limit to 4.8 mg/kg.

The highest concentrations of Cd among different food categories are detected in shellfish and molluscs, ranging from 0.01 to 4.8 mg/kg. The elevated accumulation of Cd in shellfish and molluscs is attributed to their greater ability to accumulate Cd compared to other seafood, since Cd occurs in trace concentrations in the marine environment [43, 44]. However, Cd concentrations in fish and molluscs can vary considerably between countries. For example, data from the Japanese Total Diet Study conducted during 2013–2018 revealed that Cd concentrations in fish and shellfish ranged from 0.005 to 0.12 mg/kg [25]. In a comprehensive survey conducted in China, the mean Cd concentrations in 21,392 fish and molluscs ranged from 0.007 mg/kg to 0.38 mg/kg [45]. Studies have demonstrated that differences in pH and seawater chemistry can influence the absorption, uptake, and toxicity of Cd in seawater, thereby leading to variations in Cd accumulations in shellfish and molluscs [46, 47].

Relatively high concentrations of Cd (0.0001–1.8 mg/kg) have been reported in cocoa, tea, and coffee (Table 1**)**. Cocoa based products, such as chocolate, are widely consumed globally, and some of these products have been found to contain increased levels of Cd [48–51]. Several factors may contribute to the high Cd concentration in cocoa products. For example, in some South American countries, elevated Cd concentrations in cocoa beans are attributed to naturally occurring high levels of Cd in the soil [52]. Additionally, Zug et al. [53] revealed that the use of fertilizers significantly increase Cd concentrations in cocoa products, with Cd concentrations being 1.8 mg/kg without fertilization, increasing to 3.5 mg/kg after the application of fertilizers. Therefore, in order to reduce the risk of Cd exposure through cocoa products, the European Food Safety Authority (EFSA) enacted strict regulations for maximum Cd in different cocoa products based on the risk assessment of Cd exposure in the European Union [54]. Since January 2019, the maximum Cd levels of 0.3 mg/kg have been set for chocolate with ≥ 30% and < 50% of total dry cocoa solids.

Additionally, elevated levels of Cd are also found in offal products (0.03–0.5 mg/kg), such as liver and kidney. Other food items with relatively high Cd levels include spices (0.006–0.2 mg/kg), nuts and oilseeds (0.02–0.1 mg/kg), and vegetables (0.006–0.1 mg/kg). The Total Diet Study conducted in Japan (0.004–0.08 mg/kg), China (0.014 mg/kg), and France (0.01 mg/kg) reported similar Cd concentrations in vegetables compared to those found in this study [24, 25, 55]. Plant uptake of Cd depends on several factors, of which the types of vegetables, Cd concentrations in soil and soil pH are the most important [56, 57]. Among vegetables, leafy vegetables such as spinach and lettuce tend to accumulate higher concentrations of Cd than rootstalk and legume vegetables [56]. Similarly, a large survey conducted in China found that leafy vegetables had a relatively higher Cd concentration of 0.02 mg/kg compared to other types of vegetables (0.007–0.015 mg/kg) [45]. The accumulation of Cd in vegetables is linked to soil pH, with Cd availability increasing as soil pH decreases [58, 59]. Therefore, understanding the key factors influencing Cd uptake is crucial for minimizing Cd accumulation in vegetables.

Rice is the staple food for more than half of the global population. For these populations, rice is also the most important dietary source of Cd exposure [60, 61]. National mean Cd concentration in rice varies among countries, ranging from 0.004 to 0.02 mg/kg (Table 1), well below the current limits set by FAO/WHO (0.4 mg/kg) and the European Union (0.15 mg/kg). The data is similar to that reported in a large scale survey conducted across 32 countries, revealing national median Cd concentrations between 0.005 and 0.09 mg/kg [62]. However, Cd concentrations in rice exhibits significant variability within each country. For example, Cd concentration in rice from India varied from 0.005 mg/kg to 1.005 mg/kg [62]. Similarly, a national survey in China indicated that Cd concentrations in 712 types of market rice varied from < 0.001 mg/kg to 0.74 mg/kg, with 2.2% surpassing China’s maximum limit of 0.2 mg/kg [63]. The wide range of Cd concentrations in rice can be attributed to geographic, genetic, and processing factors. For example, in 1,763 rice accessions of diverse geographic and genetic origin, accumulation of Cd in rice showed 41 and 154 times variation under flooded and non-flooded conditions, respectively [64]. In South China, an examination of 471 locally adapted high-yielding rice cultivars revealed Cd concentrations differing by 10–32 fold at different field sites [65]. Additionally, polishing brown rice to white rice can typically reduce Cd concentration by 10–30%, although in some cases polishing may increase Cd concentration in white rice [66, 67].

### Lead in Food

In comparison to As and Cd, Pb concentrations in food from different countries are generally lower, ranging from below the detection limit to 1.03 mg/kg (Table 1**)**. Among different food categories, higher national mean Pb concentrations are reported for coffee, tea, and cocoa (not detected–1.03 mg/kg), vegetables (not detected–0.4 mg/kg), alcoholic beverages (not detected–0.38 mg/kg), and beverages (not detected–0.35 mg/kg). Besides the uptake and translocation of Pb from soil, atmospheric deposition and foliar absorption may also contribute to the accumulation of Pb in the edible parts, including rice grains, carrot, and celeriac [68, 69]. Large foliage area and elevated Pb solubility in acid tea garden soils may lead to the higher accumulation of Pb in tea compared to other foods [70]. Alcoholic beverages containing high Pb concentrations may be due to the use of lead arsenate as pesticide in vineyards. Furthermore, Pb contamination of containers, such as glass bottles or metallic capsules used to seal alcoholic beverage bottles could also contribute to elevated Pb in alcoholic beverages [71].

The mean Pb concentrations in other foods are not detected–0.029 mg/kg in cereals, not detected–0.22 mg/kg in fish, not detected–0.004 mg/kg in rice, and not detected–0.009 mg/kg in wheat. However, various surveys conducted on rice samples from different countries have shown a wide range of Pb concentrations. For example, in a large-scale survey conducted across 13 countries, Pb concentrations in 782 market rice samples (0.001–0.33 mg/kg) were found to be higher compared to the Pb concentrations observed in this study [72]. Similarly, Qian et al., [63] reported that Pb concentrations in rice ranged from < 0.005 mg/kg to 0.4 mg/kg in 712 milled rice samples in China, with 0.84% of the samples exceeding the current maximum limit of 0.2 mg/kg. The lower accumulation of Pb in food crops compared to As and Cd could be attributed to the lower solubility of Pb in soil. In addition, Pb is strongly absorbed on root surfaces or chelated in root cells, limiting its translocation from roots to the above-ground edible parts [73, 74].

However, in Pb contaminated areas, relatively high concentrations of Pb were found in food samples [72, 75]. In 11 mining-impacted areas in Hunan province in China, median Pb concentrations ranged from 0.05 mg/kg to 0.78 mg/kg [75]. In an e-waste recycling area in southeast China, the mean Pb concentration in rice grain was up to 2.04 mg/kg [76].

### Dietary Exposure to Toxic Metals/Metalloids

Dietary intake of metals/metalloids depends not only on their concentrations in food, but also on food consumption patterns. For example, foodstuffs with high Cd concentrations, such as aquatic foods, may make a minimal contribution to overall Cd intake if consumed in small quantities (Fig. 1C). Similarly, in the case of Pb exposure, even though Pb concentrations in cereals are low, a high rate of consumption could significantly contribute to dietary Pb intake (Fig. 1D). Therefore, to accurately assess the potential health effects of metal/metalloid exposures through food consumption, it is important to estimate the dietary intake of these toxic metals/metalloids.

### Dietary Intake of As

**Fig. 2:**
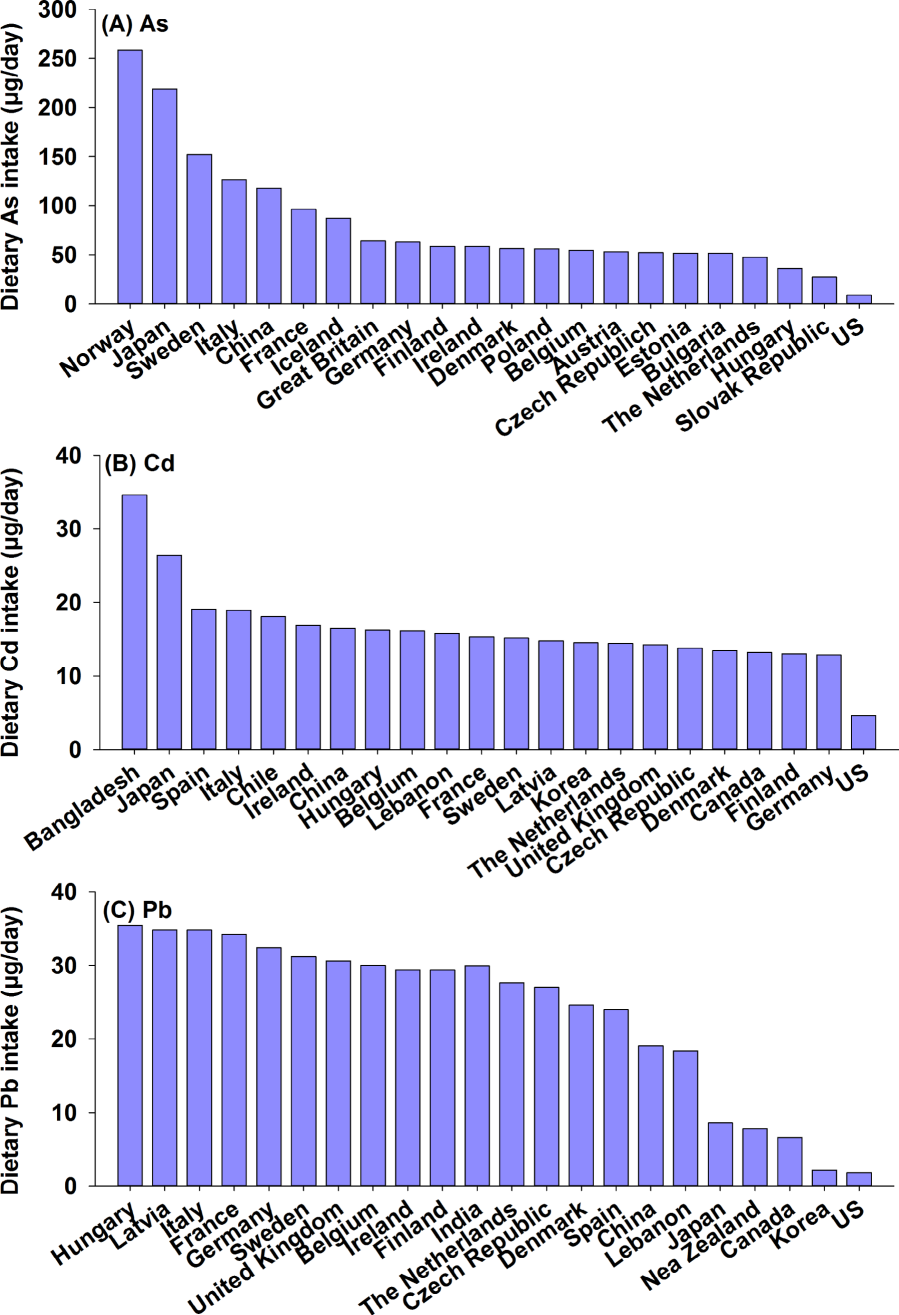
Dietary intake of toxic metals/metalloids in different countries (data obtained from [16, 24, 25, 77, 78, 81, 88–91, 141, 142]

Dietary intake of total As varies widely by country, ranging from 9 µg/day to 258.4 µg/day (Fig. 2A). In 19 European countries, the average estimated dietary As intake during 2003–2008 range from 26.9 µg/day to 258.4 µg/day, based on a total of 100,867 analytical results for As in food and drinking water, as well as the consumption data from different dietary surveys [77]. Among these countries, higher dietary As intake are reported for Norway (258.4 µg/day), Sweden (152.1 µg/day), and Italy (126.8 µg/day), while lower dietary As intake is observed in Hungary (35.9 µg/day) and Slovak Republic (26.9 µg/day). The variation in dietary As intake between different countries is caused by different consumption patterns, since average As concentrations in different food categories at European level is used for calculation. For the general population in Japan, dietary As intake was 219 µg/day based on Total Diet Study conducted during 2013–2018 [25]. For adults in China, dietary As intake was 118.1 µg/day based on the 5th Total Diet Study [24]. While for the general US population, their dietary exposure to As was the lowest (9 µg/day) compared to other countries, as indicated by available data from the FDA’s Total Diet Study (2006–2008) [78]. However, dietary intake values change over time due to changes in dietary structure and variations in contaminant concentrations in different countries. For example, the Total Diet Study reported that the average dietary intake of As in China increased from 62.3 µg/day to 118.1 µg/day during the period of 2007 to 2009–2013 [24]. Therefore, caution should be paid when interpreting these data.

Among different food categories, aquatic food products contribute the most to total As intake (44.3%). However, the risk associated with consuming aquatic food is relatively low due to the predominance of less toxic organic As species in these food (Fig. 1A and B). Consequently, more attention has been paid to the health effects of iAs exposure due to the higher toxicity compared to organic As. To protect against adverse health effects of iAs exposure, the EFSA Panel on Contaminants in the Food Chain (CONTAM Panel) established a benchmark dose lower confidence limit (BMDL_01_) of 0.3–8 µg/kg bw/day (18–480 µg/day) for a 1% increased risk of lung, skin, bladder, and skin lesions (Table 2) [77]. In 2010, the JECFA identified a BMDL_05_ of 3.0 µg/kg bw/day (180 µg/day) for a 0.5% increased incidence of lung cancer based on epidemiological studies using a range of assumptions to estimate dietary iAs exposures from drinking water and food [79].

**Table 2:** Health based guidance values (HBGV) or benchmark dose lower confidence levels (BMDLs) of metals/metalloids

Metals/metalloids	JECFA				EFSA		
	HBGV	Effect	Reference		HBGV	Effect	Reference
Inorganic arsenic	BMDL_0.5_: 3 µg/kg bw/day	0.5% increase incidence of lung cancer.	[143]		BMDL_01_: 0.3–8 µg/kg bw/day	1% increase in risk of lung, skin and bladder cancer, as well as skin lesions.	[77]
Cadmium	PTMI: 25 µg/kg bw/month	Kidney toxicity, renal proteinuria.	[144]		TWI: 2.5 µg/kg bw/week	Kidney toxicity, renal proteinuria.	[145]
Lead	Previous PTWI withdrawn	No evidence for a threshold for critical Pb induced effects.	[144]		BMDL_01_: 0.50 µg/kg bw/day	Developmental neurotoxicity	[101]
					BMDL_01_: 1.50 µg/kg bw/day	Systolic blood pressure	
					BMDL_10_: 0.63 µg/kg bw/day	Chronic kidney disease	

JECFA: Joint FAO/WHO Expert Committee on Food Additives; EFSA: European Food Safety Authority; PTMI: provisional tolerable monthly intake; PTWI: provisional tolerable weekly intake; TWI: tolerable weekly intake. BMDL_0.5_: 95% lower confidence limit on the benchmark dose for a 0.5% response; BMDL_01_: 95% lower confidence limit on the benchmark dose for a 1% response; BMDL_10_: 95% lower confidence limit on the benchmark dose for a 10% response

The average iAs intake in the European population, as assessed by EFSA, ranges from 5.4 to 27 µg/day [80], which is below the BMDL_05_ set by the JECFA. Among different food groups, grain-based products is the main contributor (23%) to iAs intake [80]. Similarly for Korean adult, the daily intake of iAs was estimated to be 10.4 µg/day, with grains (61.5%) and seaweeds (19.2%) contributing the most to dietary iAs exposure [81], highlighting the important role of grain consumption to the overall iAs exposure. Meharg et al. [8] estimated iAs intake from rice (1.2–36.2 µg/day) for 5 countries, with the associated excess cancer risk varying from 0.7 per 10,000 for Italians to 22 per 10,000 for Bangladeshis. Nunes et al. [82] estimated the excess lifetime risk of iAs exposure from rice consumption for 153 countries and found that Bangladesh (150 per 100,000) had the highest excess lifetime risk, followed by Vietnam (141 per 100,000) and Cambodia (111 per 100,000). Therefore, to reduce human exposure of iAs through dietary intake, more attention should be paid to those food categories that contribute most to overall iAs exposure, particularly grain-based products.

Moreover, recent assessments of chronic iAs exposure in European populations indicate that dietary iAs intake varies among different age groups, with children consuming 2–3 times more iAs than adults [80]. In addition to being the most highly exposed age groups, children are more sensitive to the toxicity of As, leading to an increased risk of neurodevelopment and behavioral disorders, as well as adverse immune responses, even at relatively low levels of exposure [83, 84]. Similarly, Signes-Pastor et al. [85] found that post-weaning infants consuming rice-based products had 1.6–times higher urinary iAs concentrations compared to pre-weaning infants, highlighting the potential health risks associated with rice consumption containing iAs. Additionally, several studies indicate that men are more susceptible to As-related health effects than women [86, 87]. In a population-based study conducted in Matlab, Bangladesh, men experienced twice the risk of As-related skin lesions compared to women [86]. This difference is partly due to variations in As metabolism and excretion between genders, with As methylation being less efficient in men [87].

### Dietary Intake of Cd

Dietary Cd intake varies largely across countries, ranging from 4.6 µg/day to 34.6 µg/day (Fig. 2B). The highest exposure was reported for Bangladesh, with daily dietary Cd intake being 34.6 µg/day during 2008–2009 [88]. In Europe, by using Cd occurrence data for all food categories from 22 European countries and consumption data from 32 different dietary surveys covering more than 67,000 individuals, the estimated intake varied from 12.9 µg/day to 19.1 µg/day [89]. Among different food categories, cereals (26.6%), vegetables (17.4%), and starchy roots and tubers (12.3%) are the main contributors to overall Cd intake. In Asian countries, the average dietary Cd intake was 18.0 µg/day in Japan and 32.7 µg/day in China, based on the Total Diet Study conducted during 2013–2018 and 2009–2013 [24, 25]. Compared to European populations, cereals contributed higher percentages (up to 49–65.4%) to dietary Cd intake in Asia countries [24, 25]. In Canada, the median dietary Cd intake was 18 µg/day for males aged 51–71, based on occurrence data from 2009 to 2015 [90]. The lowest dietary Cd intake of 4.6 µg/day was observed in the US, based on data from the Total Diet Study 2006–2013 and NHANES 2007–2012 [91]. The primary food categories contributing to Cd exposure were cereals and bread (34%), leafy vegetables (20%), and potatoes (11%) [91]. Satarug et al. [92] found similar Cd intake values of 8–25 µg/day in adults across different countries. Most countries’ Cd intake values are below the provisional tolerated weekly intake (PTWI) of 50 µg/day set by the JECFA and 21.4 µg/day set by EFSA for a body weight of 60 kg. However, the only exception is Bangladesh, where the dietary Cd intake exceeds the limit of 21.4 µg/day set by EFSA, primarily due to higher consumption rates of rice and vegetables, and lower consumption rates of animal products [88].

Dietary Cd intake varies considerably across different age groups. For example, dietary Cd intake is highest in toddlers for European populations with an average intake of 41.6 µg/day, but lowest in the elderly population (13.4 µg/day) [89]. The higher intake of Cd in toddlers is mainly due to the higher food consumption rate compared to adults at young ages. Additionally, evidence indicates that women may be more adversely affected by Cd than men, particularly regarding bone health, as women may retain higher levels of Cd in their body tissues compared to men [93, 94]. Moreover, the most severe disease of Cd toxicity, known as itai-itai disease, a combination of kidney damage, osteoporosis, and osteomalacia, is notably more prevalent in women compared to men [95].

However, epidemiological studies from various countries suggest that exposure to Cd at levels lower than the PTWI set by EFSA or JECFA could also contribute to several adverse health outcomes. For Japanese women, dietary Cd intake exceeding 31.5 µg/day was linked to an elevated risk of estrogen receptor positive breast cancer [96]. Similarly, in a study by Julin et al. [97] involving 41,089 Swedish men with a mean dietary Cd intake of 19 µg/day, multivariable-adjusted dietary Cd intake was positively associated with a higher incidence of overall prostate cancer [97]. Therefore, there is no margin of safety between Cd exposure levels and benchmark dose levels for possible adverse health effects, especially for vulnerable populations.

### Dietary Intake of Pb

**Fig. 3:**
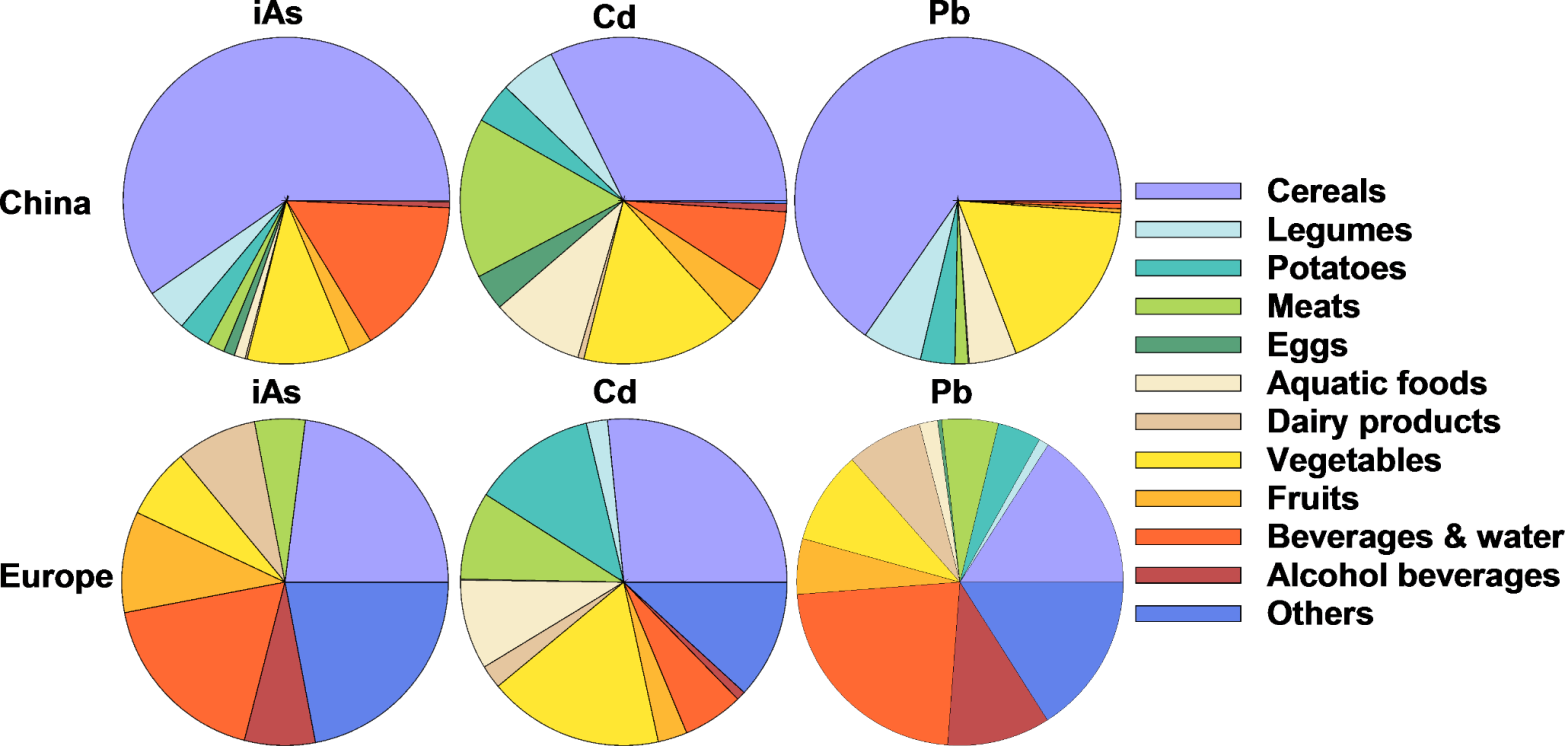
Contributions of different food categories to overall metal exposure in China and Europe (data obtained from [24, 80, 89, 98])

The average dietary Pb intake varies from 1.8 to 35.4 µg/day among different countries (Fig. 2C). In 14 European countries, dietary Pb exposure ranged from 24 to 35.4 µg/day, based on Pb occurrence data in 144,206 foods coupled with a total of 32 dietary surveys covering more than 67,000 individuals [98]. Among different European countries, the average dietary Pb intake is highest in Hungary (35.4 µg/day), while lowest in Spain (24 µg/day). The reported dietary Pb intake for other countries are 29.9 µg/day in India, 19.1 µg/day in China, 8.6 µg/day in Japan, 6.6 µg/day in Canada, and 1.8 µg/day in the US. The variation in dietary Pb intake among different countries may be linked to their primary sources of exposure. For the Chinese population, the major contributors to Pb intake are cereals (32.3%), meats (15.9%), and vegetables products (15.6%), together contributing 64% to the overall dietary Pb intake [24]. While for Europeans, the food categories with major contributions are beverages and water (22.6%), grain and grain products (15.9%), alcoholic beverages (10.2%), and vegetables products (9.2%), which all together contribute 58% to the dietary intake [98] (Fig. 3).

Furthermore, gender differences greatly impact the adverse health effects of Pb exposure. Studies indicate that males tend to show more pronounced cognitive, behavioral, and respiratory changes, whereas females are more susceptible to chemoreflex hypersensitivity [99, 100]. This emphasizes the importance of considering gender differences in toxicity when assessing potential health effects.

Compared to the health based guidance values (HBGVs) set by different organizations, all countries have dietary Pb intake lower than the limits for extra-risk of systolic blood pressure (1.50 µg/kg bw/day) and chronic kidney disease (0.63 µg/kg bw/day). However, in some European countries, such as Hungary, Latvia, Italy, France, Germany, Sweden, and the United Kingdom, dietary Pb intake exceeds the benchmark dose of 1% extra-risk for developmental neurotoxicity (0.5 µg/kg bw/day) [101]. Emerging evidence suggests that chronic Pb exposure, even at levels lower than HBGVs is associated with increased risk of intellectual impairment and osteoporosis [102, 103], leading to growing concerns for Pb exposures at lower levels which were previously assumed to be safe. Therefore, to protect humans from Pb exposure, especially for vulnerable populations such as fetuses, infants, and children, more stringent HBGVs and intervention strategies should be developed. Furthermore, the current exposure to Pb only considers dietary intake. Children inhalation/ingestion of soil and dust can be an important contributor of Pb exposure. Therefore, the assessment of Pb exposure through other pathways should be taken into account.

### Strategies to Reduce Metals/Metalloids Exposure

To protect humans from metals/metalloids toxicity via dietary intake, measures can be taken throughout the food chain, including reducing metals/metalloids input to the environment through anthropogenic sources, reducing metals/metalloids accumulation in food through agricultural practices, developing new crop varieties with reduced accumulation of toxic metals/metalloids through selective breeding techniques, and changing diet structure to reduce metal/metalloid intake.

### Reduction of Metals/Metalloids Input to the Environment from Anthropogenic Sources

Besides geogenic sources, metal/metalloid contamination in the environment is mainly attributed to anthropogenic activities, including mining, agriculture, and industry sources. Elevated metal/metalloid concentrations in soil, water, and air may occur in areas close to mining, smelting and refining of nonferrous metals/metalloids industries. Arsenic is widely distributed in the Earth crust, as the by-product of smelting copper, lead or gold ores. Cadmium rarely exists in the environment as a pure metal, but primarily occurs in Zn or Pb ores in relatively high concentrations as by-product of the refining of Zn, Cu, and Pb [104]. Lead ore processing has resulted in extensive release of Pb into the environment. It was estimated that ~ 300 million tons of Pb was released into the environment within the past 500 years due to global lead ore processing [10]. Elevated metal/metalloid concentrations in environmental matrices have been reported due to mining/smelting activities, especially in soils, therefore resulting in elevated metals/metalloids in food [105–107]. A global survey estimated that 23 million people living on floodplain are affected by toxic waste derived from past and current mining activities [108]. China and the US are the most affected countries, with 9.74 million and 3.17 million people being affected, respectively [108].

Besides mining/smelting activities, unintentional agricultural practices, such as the use of metal-containing pesticides and fertilizers are also a potentially important source of metal/metalloid contamination in food. Arsenic compounds such as roxarsone and nitarsone have been used in poultry production for years to treat coccidiosis and promote growth, leading to elevated As in chicken meat [109–111]. Even though some forms of these As-containing pesticides have been phased out in countries including the US, Canada, Europe, and China, these pesticides are still being used in many other countries, resulting in As contaminated food [109, 111]. Furthermore, application of phosphate fertilizers has resulted in elevated Cd in soil, since phosphate fertilizers are produced from phosphate rocks, which contain Cd as a natural contaminant. Nziguheba and Smolders [112] collected 196 phosphate fertilizers from 12 European countries, with the concentrations of Cd being < 0.2–275 mg/kg. Therefore, annual application of 30 kg/ha those phosphate fertilizers can introduce < 6–8250 mg/ha Cd into the soil.

Irrigation of paddy soils with metal/metalloid contaminated water can also lead to the build-up of metals/metalloids in soil, thereby elevating metal/metalloid accumulation in plant foods. Arsenic contamination of groundwater has been widely reported in South and Southeast Asian countries, and increasing evidence showed that soil As levels have increased as a result of irrigation using contaminated groundwater [113, 114]. It was reported that a soil irrigated with water containing 0.1 mg/kg As for a year can add 0.56 mg/kg As to soil [19]. Rice from districts irrigated with As contaminated water (> 50 µg/L) is significantly higher than rice from uncontaminated districts in Bangladesh [114]. Moreover, in an area of Beijing, China, farmland irrigation with wastewater has led to an 84–fold increase in Cd concentrations and an 18–fold increase in Pb concentrations over the past 30 years [115]. Therefore, avoiding inadequate treatment of wastewater or using alternative clean water for irrigation could significantly reduce metal/metalloid accumulation in food [116, 117].

Atmospheric deposition is a significant source of metal/metalloid contamination in soil. For example, in a study conducted in England and Wales, atmospheric deposition contributed to 56%, 53%, and 78% of As, Cd, and Pb contamination in the whole study area [118]. Specifically, atmospheric contamination of Pb from Pb-based gasoline or industrial pollution has been the primary source of Pb in crop plants, since the bioavailability of Pb in soil is low and the mobility of Pb in plants is limited [73, 74]. The phase-out of Pb in gasoline has been effective in reducing Pb exposure [119]. Therefore, restricting Pb emissions from industry could also contribute to reduced Pb levels in food.

### Reduction of Metals/Metalloids in Food through Agricultural Practices

Agricultural practices such as liming, which uses calcium carbonate to ameliorate soil acidity, and the application of biochar, a charcoal-like material produced by burning organic agricultural waste, can effectively reduce the bioavailability of Cd and Pb in soil. Additionally, careful management of water irrigation regimes to control As and Cd bioavailability can greatly affect metal/metalloid accumulations in food crops.

The bioavailability and accumulation of metals/metalloids in crop plants are strongly influenced by soil pH. For example, the solubility of Cd in soil increases by 3.5 times with one-unit decrease in soil pH [120]. Therefore, liming is an effective method for reducing the availability of metals/metalloids, especially for Cd and Pb. A single application of CaCO_3_ at 7.5 ton/hectare increased soil pH from 5.5 to 6.5 and decreased grain Cd concentration by 70–80%, with no negative effects on grain yield [121]. In a three-year field study conducted in southern China, single soil amendment of wheat straw biochar in Cd and Pb co-contaminated paddy field reduced Cd and Pb in rice grain by 28–67% and 27–69%, respectively [122]. While liming had no significant effect on As in rice grain [123].

Water management plays an important role in reducing metals/metalloids accumulation in plants by influencing soil redox potential, especially for As and Cd. Generally, Cd is more available in aerobic soils than in anaerobic soils because of the formation of insoluble CdS. In contrast, flooding of paddy soil increases As availability due to reductive dissolution of iron oxyhydroxides and reduction of arsenate to more mobile arsenite [124–126]. Both the greenhouse and field studies indicate that cultivating rice in aerobic soil can reduce As concentrations in rice grains, but lead to increased Cd concentration [126, 127]. However, because the effects of water management on As and Cd availability in paddy soil are opposite, the options for water management depend on the relative risk of As and Cd accumulation for soils co-contaminated with As and Cd.

### Breeding Crop Cultivars with Low Accumulation of Toxic Metals/Metalloids

Great efforts have been made through breeding crop cultivars with low accumulation of toxic metals/metalloids, especially for As and Cd. A number of genes responsible for As and Cd uptake in rice have been identified [128]. For example, *OsNRAMP5* and *OsHMA3* are two genes that play a crucial role in controlling Cd uptake and translocation in rice plant [129, 130]. Mutation of *OsNRAMP5* decreased Cd concentration in rice by > 95% [131], and also decreased Pb concentration in rice grain by 50% [132]. Mutants of *OsNRAMP5* generated by chemical or physical mutations have been used to breed rice cultivars with very low accumulation of Cd. Overexpression of *OsHMA3* in rice greatly decreased accumulation of Cd in grain, without affecting other micronutrients in the grain [129, 133]. Besides rice, several quantitative trait loci (QTLs) controlling As and Cd accumulation in edible tissues of different crops were also detected, such as wheat, barley, and potato tubers [134–136]. For example, Cd accumulation in durum wheat is governed by the major QTL *Cdu1* [134]. This information was used to breed low Cd durum wheat cultivars by using a molecular marker for the *Cdu1* QTL. Moreover, overexpression of the *OsHMA3* gene in wheat decreased Cd accumulation in wheat grains by 96% [137].

### Changing Diet Structure to Reduce Metal/Metalloid Intake

Cereals are the major source of metal/metalloid exposure, especially for Asian populations. If the dietary structure overly relies on some cereals, such as rice and wheat which have low concentrations of Fe and Zn, may not only fail to provide sufficient levels of these essential nutrients, but also could increase the absorption of metals/metalloids in the human body due to the competition with toxic metals/metalloids [138, 139]. Therefore, a well-balanced diet rich in mineral nutrients, vitamins, and amino acids is essential for reducing human dietary exposure to toxic metals/metalloids.

Edible seaweeds with a relatively high As concentration have been a global concern, especially for populations in Korea, Japan, and China. Sensitive method for the detection of As speciation in seaweed is beneficial for estimating the dietary exposure of As in humans. To minimize the food chain As exposure, lowering the consumption rate of seaweed is highly recommended [140].

## Conclusion

Food contamination with metals/metalloids poses significant concerns for human health, particularly for vulnerable populations. Dietary intake of metals/metalloids is depended not only on food concentrations of metals/metalloids, but also the amount of the food consumed and As species present in food items. Even though significant progress has been made in understanding of the current status of metal/metalloid contamination in different food categories and their related health risks, there are still many knowledge gaps: (1) Exposure profiles using average metal/metalloid concentrations in food may be misleading, since there are still many hot spots suffering from metal/metalloid contamination due to anthropogenic activities such as mining/smelting. Therefore, the body burden data of toxic metals/metalloids for humans with potentially high exposures in contaminated areas are urgently needed. (2) The potential adverse health effects of metal/metalloid exposure via dietary intake for vulnerable populations, especially for infants, pregnant women, and the elderly should also be fully investigated. (3) More consideration of the toxicity of some organic As species is needed for understanding the potential health hazards, such as methylated As species which are commonly detected in seafood or new detected As species such as DMMTA in rice.

## Data Availability

No datasets were generated or analysed during the current study.
